# Histological Changes in the Blood Vessels of Ruptured Human Anterior Cruciate Ligaments

**DOI:** 10.7759/cureus.68989

**Published:** 2024-09-09

**Authors:** Jakkula Akhil, Yogesh Sontakke, Gopisankar Balaji

**Affiliations:** 1 Anatomy, Pondicherry Institute of Medical Sciences (PIMS), Puducherry, IND; 2 Anatomy, Jawaharlal Institute of Postgraduate Medical Education and Research, Puducherry, IND; 3 Orthopaedics, Jawaharlal Institute of Postgraduate Medical Education and Research, Puducherry, IND

**Keywords:** angiogenesis, blood vessel density, healing potential, location of blood vessels, luminal area, ruptured anterior cruciate ligament

## Abstract

Background: Anterior cruciate ligament (ACL) rupture is a commonly encountered sports injury worldwide. ACL rupture is known to have poor healing capacity, hypothesized to be due to low vascularity. ACL reconstruction surgery with ligament removal and tendon graft became essential for the higher grades of ACL tears. However, ACL-reconstructed patients faced post-traumatic osteoarthritis 10-15 years after surgery. In the recent past, the tibial remnant of ACL was shown to have intrinsic healing potential. Blood vessel density and the location of blood vessels of ACL remnants have critical implications in the newly upcoming remnant-preservation ACL reconstruction surgeries that showed better healing response. This study was performed to characterize the histological features of ruptured ACL remnants in terms of blood vessels to assess the healing potential and their utility in novel surgical techniques.

Methods: This was a descriptive cross-sectional study in which the tibial remnant of 24 ruptured ACL samples was evaluated for blood vessel density (per sq. mm), luminal area (sq. µm), and location of blood vessels using hematoxylin and eosin (H&E) staining with ImageJ software (U. S. National Institutes of Health, Bethesda, Maryland, USA). The blood vessel density and location of blood vessels were compared among various groups based on the duration of injury and number of injuries.

Results: Twenty-three male and one female adult patients with a mean duration of injury of 7.54 ± 5.63 months (range: 2-24 months) were included in the study. They were divided into three groups based on duration of injury: group I (2-5 months; n = 10), group II (6-8 months; n = 8), and group III (9-24 months; n = 6). The median blood vessel density (blood vessels per sq. mm) was 5.50 (3.30, 10.23) per sq. mm. There was no correlation of blood vessel density observed with duration of injury. All groups showed similar results statistically. More patients in earlier duration of injury showed very high range (10.1-40 per sq. mm) of blood vessels compared to the patients of later duration. Immature and intermediary blood vessels were identified denoting angiogenesis. Location of blood vessels varied in the groups based on duration of injury. There was no significant difference in blood vessel density and location of blood vessels between patients with single injury and those with multiple injuries.

Conclusion: The present study demonstrates the presence of healing potential of ruptured anterior cruciate ligaments in terms of blood vessel density, luminal area, and location of blood vessels. Future studies looking into the functional outcome would enhance the understanding of utility of novel remnant-preservation surgeries in place of standard graft reconstruction surgeries.

## Introduction

Anterior cruciate ligament (ACL) is a crucial ligament in the knee joint, preventing unrestrained anterior translation and internal rotation of the tibia in relation to the femur. Histologically, ACL consists of collagen bundles with fibroblasts and minimal blood vessels. ACL is surrounded by connective tissue distinct from the ligament, termed as epiligament [1]. ACL rupture is one of the most common knee injuries worldwide. In the United States of America (USA), it constitutes more than 1,20,000 cases annually [2]. In India, John et al. found ACL injury comprising 86.5% of acute knee sports injuries [3]. It has a poor self-healing capacity, considered to be due to low vascularity [4,5]. It consists of three grades: microscopic (grade 1), partial (grade 2), and complete (grade 3) tears. Surgical treatment is required for grades 2 and 3 ACL tears. In the past, primary ACL repair was performed by suturing the ends which showed a higher failure rate [6]. The current practice and the gold standard treatment is ACL reconstruction with removal of the ligament and replacement using hamstring or patellar tendon grafts which exhibited good results [7,8]. However, 62% of ACL-reconstructed patients developed post-traumatic osteoarthritis 10-15 years after surgery [9]. There has been a recent surge of interest in remnant-preserving ACL reconstruction surgeries as it showed faster healing and better functional recovery [4,10-14]. The tibial remnant of ACL was shown to have intrinsic healing response with typical features comparable to medial collateral ligament that can heal spontaneously [7]. Therefore, we aimed to estimate the blood vessel density, luminal area of blood vessels, and predominant location of blood vessels in the tibial remnant of ACL tear to further enhance the understanding of healing potential.

This article was presented at MAHACON VI, 6th state conference of regional chapter of anatomy, Maharashtra on February 16-17, 2024.

## Materials and methods

This was a descriptive cross-sectional study conducted in the Department of Anatomy in collaboration with the Department of Orthopaedics at Jawaharlal Institute of Postgraduate Medical Education and Research (JIPMER), Puducherry, India. The study was approved by the Institute Ethics Committee for Observational Studies (JIP/IEC/2021/0126). Informed written consent was taken from the patients. The study was conducted for a period of two years from July 2021 to June 2023. Twenty-four adult patients were included in the study. All patients more than 18 years with grade 2 or 3 ACL tear undergoing ACL reconstruction for knee instability were included. Duration of injury between eight weeks and two years was included. Patients with a previous history of infection or knee joint surgery were excluded. Convenience sampling method was followed. Based on the duration of injury, patients were classified as follows: group I: 2-5 months, group II: 6-8 months, and group III: 9-24 months. Based on number of injuries, the patients were considered to have single injury and multiple injuries.

Sample collection and histology

The patients underwent arthroscopic ACL reconstruction. Tissues from the ACL remnants in the tibial footprint were collected using arthroscopic punch forceps. Epiligament, the surrounding connective tissue, was also obtained collectively. The obtained tissues were transported in 10% neutral buffered formalin to the Department of Anatomy. The tissues were fixed in 10% neutral buffered formalin, processed and embedded in paraffin as per standard histological techniques. The sections were taken at 7-10 µm thickness. The slides were stained with hematoxylin and eosin (H&E).

Micrometry

**Figure 1:**
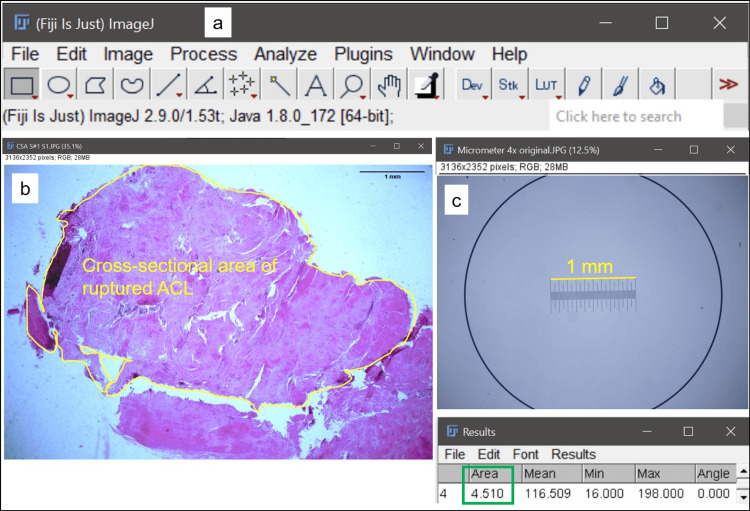
Calculation of cross–sectional area (CSA) of ruptured ACL using ImageJ a. ImageJ control panel. b. Cross-sectional area (CSA) of ruptured ACL (H&E; 4x). c. Stage micrometer showing 1 mm length with 100 divisions. Result of CSA as 4.51 sq. mm. ACL: anterior cruciate ligament, H&E: hematoxylin and eosin.

**Figure 2:**
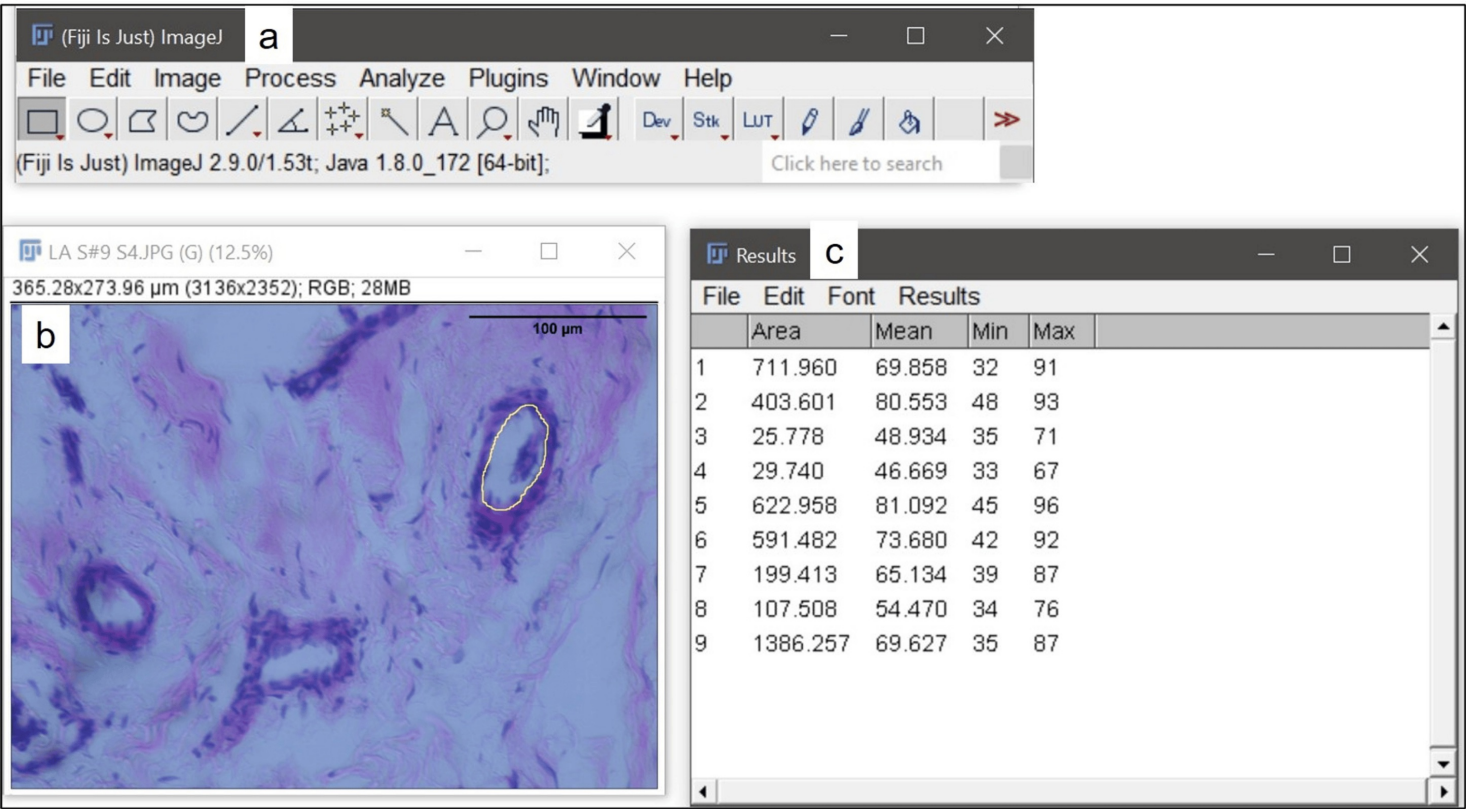
Calculation of luminal area (LA) of blood vessels in ruptured ACL using ImageJ a. ImageJ control panel. b. Luminal area calculated using freehand selections tool (H&E; 40x). c. Result of LA as 1386.257 sq. µm. ACL: anterior cruciate ligament, H&E: hematoxylin and eosin.

The slides were observed under brightfield binocular microscope Olympus CX41RF, Japan, and the images were taken with Olympus E330 camera at 4x and 40x magnification for tissue section area and luminal area, respectively. ImageJ software version 2.1.0/1.53c (RRID: SCR_003070; U. S. National Institutes of Health, Bethesda, Maryland, USA) was used for the micrometry. Stage micrometer Erma SM-001, Tokyo, was used for calibration. The tissue section area was calculated at 4x magnification (Figures 1a-1c). The luminal area was calculated at 40x magnification (Figures 2a-2c). Both the parameters were measured using the “Freehand tool”, after the global setting up of the scale using micrometer in respective magnifications.

Histological evaluation

The histological parameters studied were blood vessel density (number of vessels per sq. mm), luminal area, and location of blood vessels.

Blood Vessel Density

In H&E slides, the blood vessels were counted in the entire tissue section. The circular or longitudinal lumens lined by endothelial cells, with nuclei stained by hematoxylin along with the immature vessels lacking lumen showing vessel wall, were counted as blood vessels at 40x magnification. The number of blood vessels was divided by the tissue section area to obtain the blood vessel density (per sq. mm). The blood vessel density in samples was further arbitrarily divided into three ranges: 0-5 per sq. mm as average, 5.1-10 per sq. mm as high, and 10.1-40 per sq. mm as very high.

Luminal Area

The circular and longitudinal sections of blood vessels were measured using ImageJ "freehand selections tool". The vessels with luminal area less than 78.54 sq. µm were counted as capillaries and 78.54-7854 sq. µm were counted as arterioles or venules. The diameter of the vessels was further derived from luminal area using mathematical formulae. The vessels having lumen were considered mature vessels; the capillaries with narrow lumen as intermediary vessels; and the capillaries without lumen as immature vessels, which formed irregular clusters of nuclei in collagen bundles which stained differently from fibroblasts in collagen bundles.

Location of Blood Vessels

*​​​​​​​*The predominant location of blood vessels was ascertained whether in the epiligament or midsubstance or equally located in both. The blood vessels in the loose connective tissue surrounding the collagen bundles at the periphery of the tissue section were considered as epiligamentous blood vessels. The blood vessels amidst the collagen bundles in the center of the section were considered as midsubstance blood vessels.

Statistical analysis

Continuous variables were given as mean and standard deviation or median and interquartile ranges (IQRs), based on the normality of data (Shapiro-Wilk test). The correlation with duration of injury was performed using Spearman’s rho correlation. The comparison of three groups based on the duration of injury was performed with the Kruskal-Wallis test. The comparison of ranges of blood vessel density in three groups was performed with the Freeman-Halton extension of the Fisher exact probability test. The comparison of single and multiple injuries was performed with the Mann-Whitney U test. The comparison of three groups in single injury was done by one-way ANOVA test. The comparison of group I single and group I multiple injuries was performed with an independent t-test. The comparison of location of blood vessels was done by Freeman-Halton extension of the Fisher exact probability test. The threshold for statistical significance was considered at a p-value of less than 0.05. The statistical analyses were performed using SPSS software 19.0 (IBM Corp., Armonk, NY, USA) and Stata software 14.0 (StataCorp LP, College Station, TX, USA).

## Results

Patient characteristics

There were 23 males and one female. The mean age of the patients was 25.62 ± 5.25 years (range: 19-42 years). The mean duration of injury was 7.54 ± 5.63 months (range: 2-24 months). The patient distribution in the groups based on the duration of injury is as follows: group I: 10, group II: eight, and group III: six. Nineteen patients had a single injury, while five had multiple injuries.

Blood vessel density

**Figure 3:**
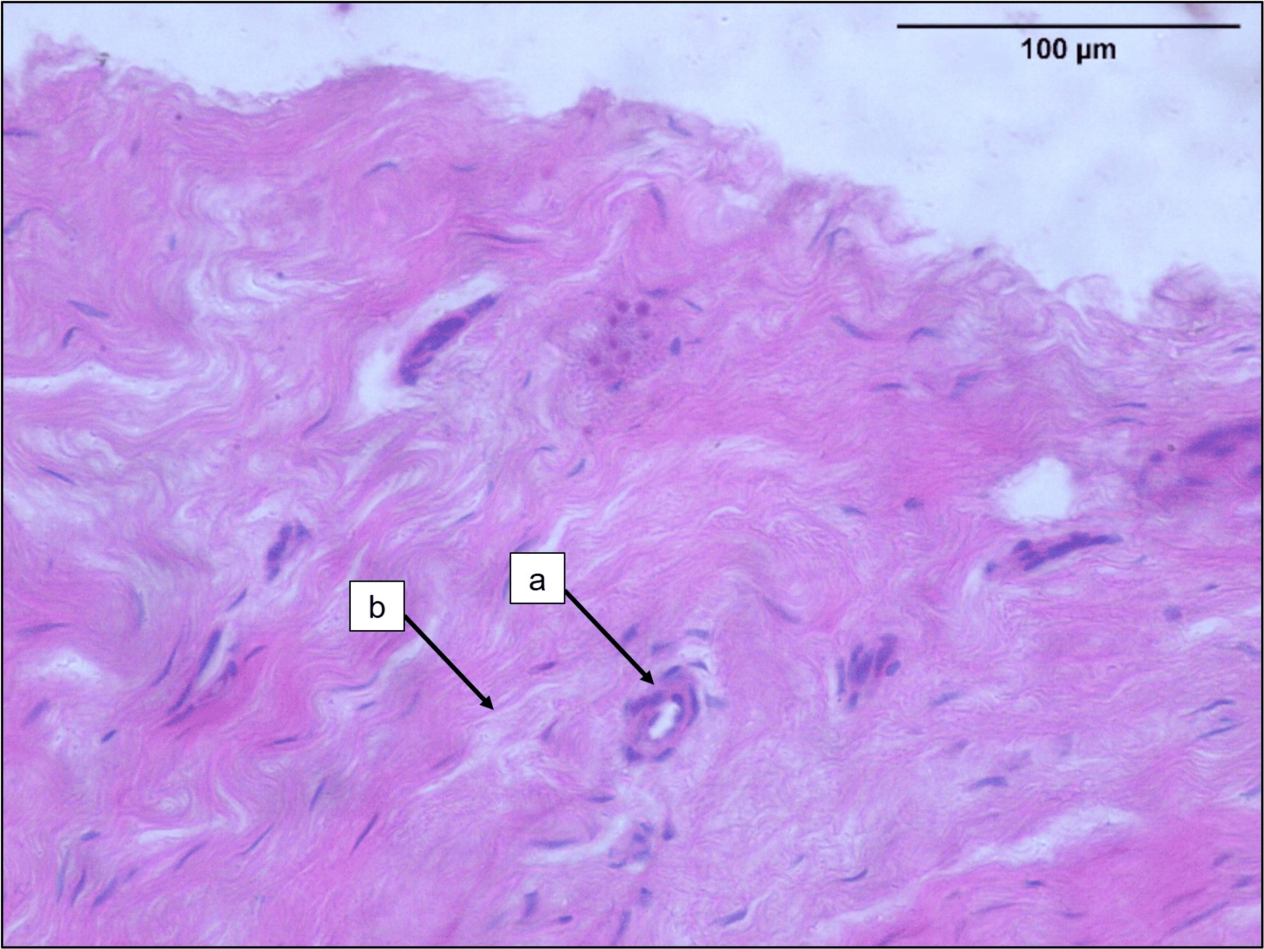
Photomicrograph of the midsubstance of tibial remnant of ruptured ACL a. Blood vessels. b. Collagen bundles (H&E; 40x). ACL: anterior cruciate ligament, H&E: hematoxylin and eosin.

**Figure 4:**
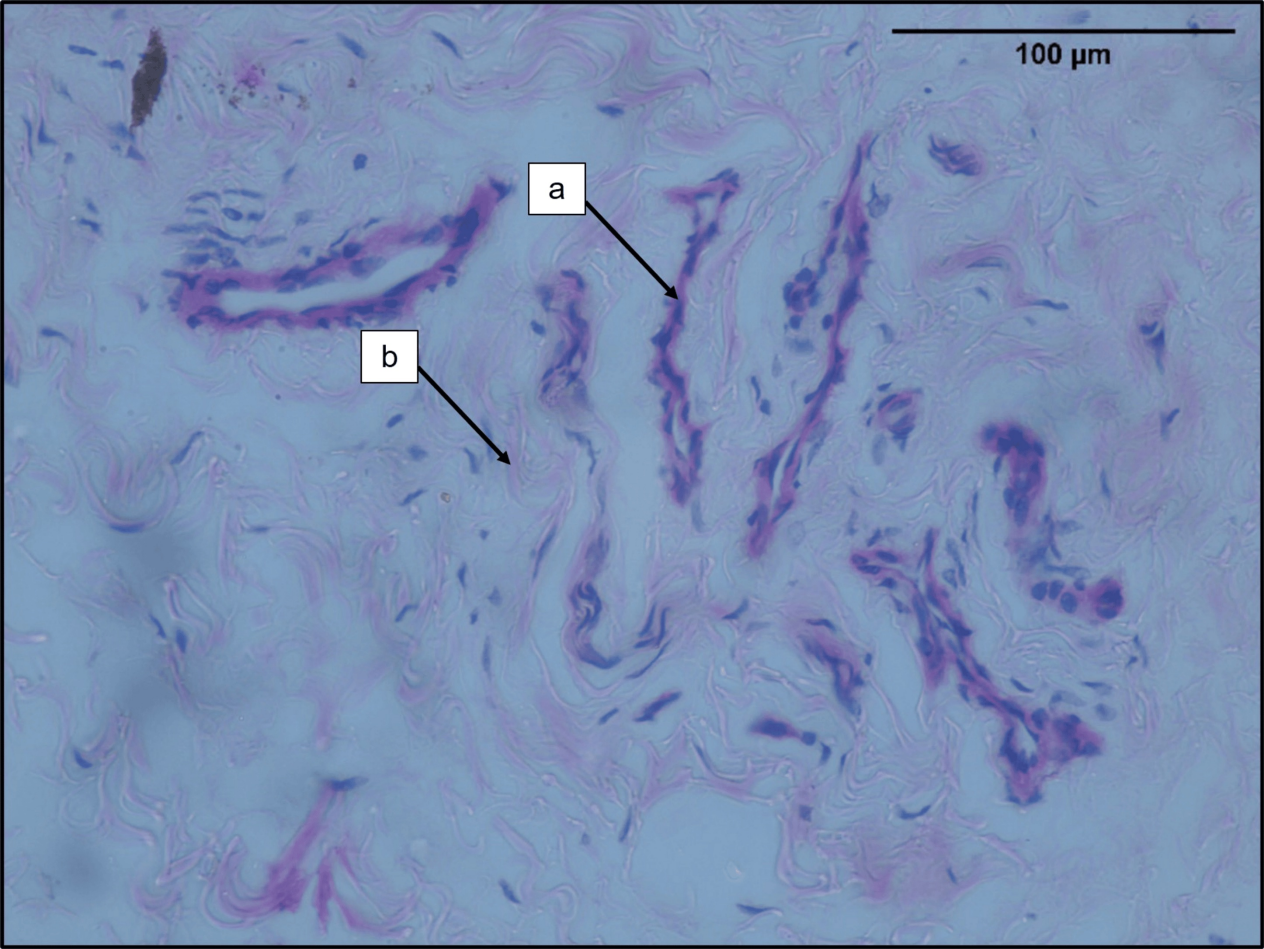
Photomicrograph of the epiligament of tibial remnant of ruptured ACL a. Blood vessels. b. Collagen bundles (H&E; 40x). ACL: anterior cruciate ligament, H&E: hematoxylin and eosin.

The blood vessels were seen in the midsubstance as well as the epiligament of the ACL sample (Figures 3, 4). The densely cellular areas showed higher number of blood vessels. The median (IQR) blood vessel density in the ruptured ACLs was 5.50 (3.30, 10.23) per sq. mm.

There was no significant correlation of blood vessel density with duration of injury. Spearman’s rho correlation was performed (r = - 0.039, p = 0.855). Comparing the three groups based on the duration of injury, there was no difference between the three groups (p = 0.885) (Table 1).

**Table 1:** Comparison of blood vessel density among three groups based on duration of injury Group I: 2-5 months, group II: 6-8 months, and group III: 9-24 months. Kruskal-Wallis test was performed. IQR: interquartile range.

Parameter	Median (IQR) (in no. per sq. mm)	p-value
Group I (n = 10)	5.43 (3.09, 11.77)	0.885
Group II (n = 8)	4.80 (3.04, 9.34)	
Group III (n = 6)	6.94 (3.84, 8.68)	

**Figure 5:**
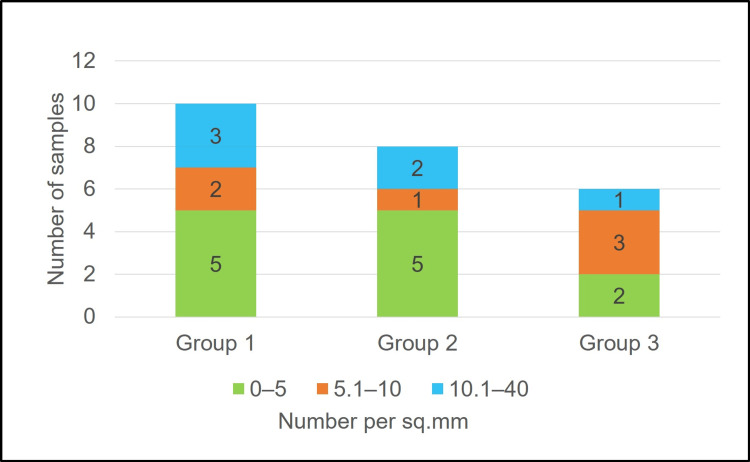
Comparison of ranges of blood vessel density among three groups based on duration of injury Group I: 2-5 months, group II: 6-8 months, and group III: 9-24 months. Freeman-Halton extension of the Fisher exact probability test was performed. p = 0.67.

The very high range (10.1-40 per sq. mm) of blood vessel density was seen in 30% of the samples in first group, 25% in the second group and 16.7% samples in the third group, but not significant statistically (p = 0.67) (Figure 5).

The comparison between single injury and multiple injuries showed that median blood vessel density in multiple injuries was twice that of single injury but not significant statistically (p = 0.374) (Table 2).

**Table 2:** Comparison of blood vessel density between single injury and multiple injuries Mann-Whitney U test was performed. IQR: interquartile range.

Parameter	Median (IQR) (in no. per sq. mm)	p-value
Single injury (n = 19)	5.00 (3.09, 8.59)	0.374
Multiple injuries (n = 5)	11.77 (2.88, 23.49)	

The comparison of three groups based on the duration of injury in single injury showed the trend of higher blood vessel density in the third group with no statistical significance (p = 0.477) (Table 3).

**Table 3:** Comparison of blood vessel density among three groups with single injury based on duration of injury Group I: 2-5 months, group II: 6-8 months, and group III: 9-24 months. One-way ANOVA test was performed. SD: standard deviation.

Parameter	Mean ± SD (in no. per sq. mm)	p-value
Group I single injury (n = 6)	4.55 ± 2.48	0.477
Group II single injury (n = 7)	7.96 ± 6.64	
Group III single injury (n = 6)	8.35 ± 7.18	

The comparison between group I single and group II multiple injuries showed recent multiple injuries having higher blood vessel density but not statistically significant (p = 0.068) (Table 4).

**Table 4:** Comparison of blood vessel density between group I single injury and group I multiple injuries Group I: 2-5 months. Independent t-test was performed. SD: standard deviation.

Parameter	Mean ± SD (in no. per sq. mm)	p-value
Group I single injury (n = 6)	4.55 ± 2.48	0.068
Group I multiple injuries (n = 4)	15.63 ± 12.93	

Luminal area

The luminal area was calculated for the blood vessels pooled from all the samples. Out of the total 280 counted blood vessels, 206 vessels had luminal area more than 78.54 sq. µm and less than 7853.98 sq. µm which constituted arterioles and post-capillary venules. The remaining 74 had less than 78.54 sq. µm which constituted capillaries (Table 5). The luminal area ranged from 3.30 sq.µm. to 1627.39 sq. µm. The derived diameter of blood vessels ranged from 2.05 µm to 45.52 µm. Seventy-four vessels had diameter less than 10 µm corresponding to capillaries.

**Table 5:** Luminal area: distribution of blood vessels

Luminal area	Derived diameter	Type of blood vessel	Number of blood vessels	Total
78.54-7853.98 sq. µm	10-100 µm	Arterioles	206	280
78.54-1963.50 sq. µm	10-50 µm	Post-capillary venules		
< 78.54 sq. µm	< 10 µm	Capillaries	74	

**Figure 6:**
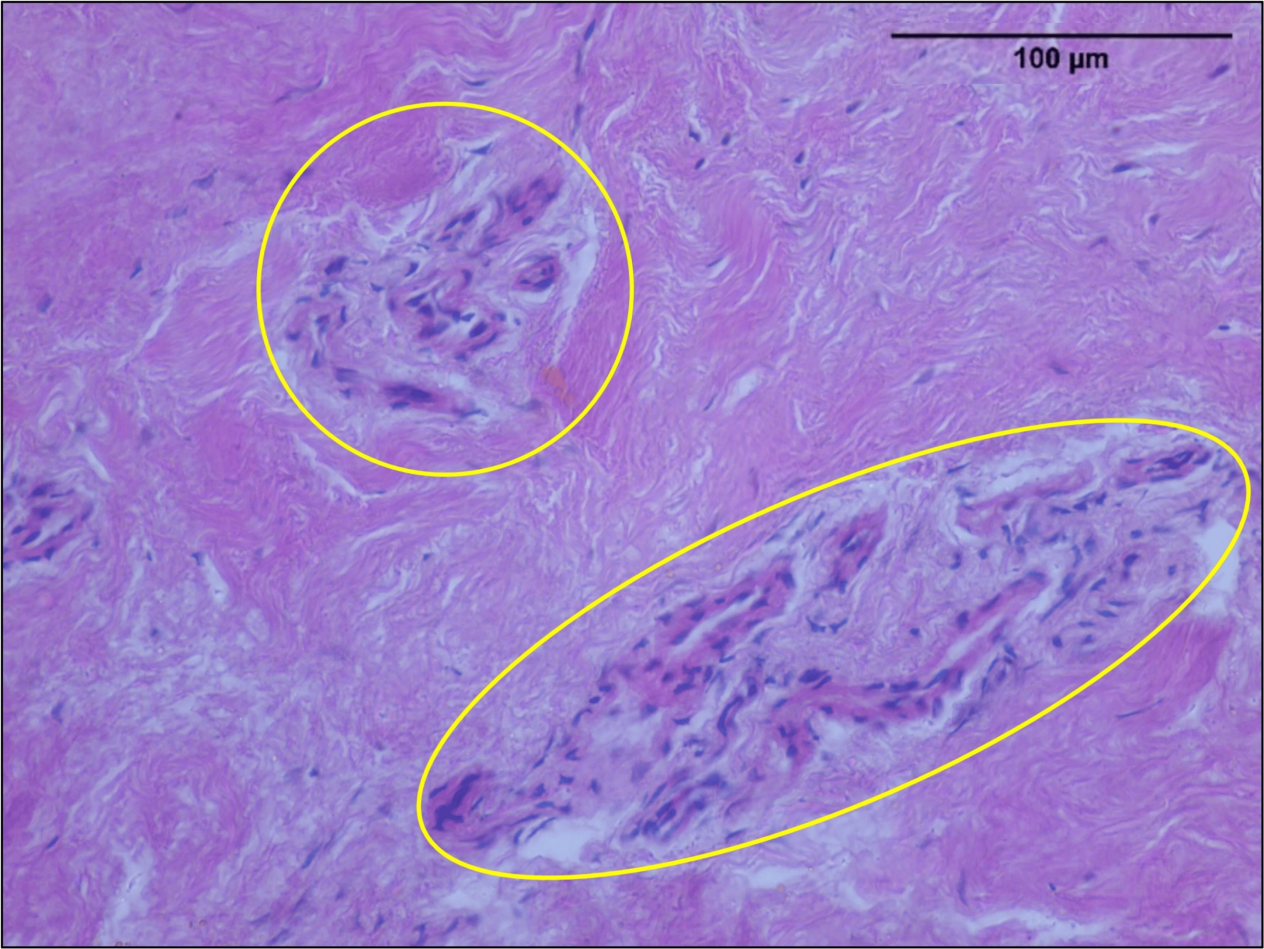
Immature and intermediary blood vessels along with the mature vessels in ruptured ACL (seen in area enclosed in yellow outlines) H&E; 40x. ACL: anterior cruciate ligament, H&E: hematoxylin and eosin.

Immature vessels with absent lumen and intermediary blood vessels with very narrow lumen were noticed alongside the mature vessels (Figure 6).

Location of blood vessels

The predominant location of blood vessels based on the duration of injury is given in Table 6. Nearly half the samples of group I had predominant location in epiligament, while the rest half of group I had predominant location in midsubstance. Group II had equally in both the locations or epiligament chiefly. Group III had mainly in the epiligament. There was no significant statistical difference (p = 0.252).

**Table 6:** Comparison of location of blood vessels among three groups based on duration of injury Group I: 2-5 months, group II: 6-8 months, and group III: 9-24 months. Freeman-Halton extension of the Fisher exact probability test was performed.

Location of blood vessels	Group I (n = 10)	Group II (n = 8)	Group III (n = 6)	p-value
Epiligament	4	3	4	0.252
Midsubstance	5	1	1	
Both	1	4	1	

The predominant location of blood vessels between single and multiple injuries is given in Table 7. Single injury and multiple injuries did not reveal any significant difference in the predominant location of blood vessels (p = 1.000).

**Table 7:** Comparison of location of blood vessels between single injury and multiple injuries Freeman-Halton extension of the Fisher exact probability test was performed.

Location of blood vessels	Single injury (n = 19)	Multiple injuries (n = 5)	p-value
Epiligament	9	2	1.000
Midsubstance	5	2	
Both	5	1	

The predominant location of blood vessels in three groups of single injury based on duration of injury is given in Table 8. Half the samples of group I had predominant location in epiligament, while the rest half of group I had predominant location in midsubstance. Group II had in both the locations or epiligament chiefly. Group III had mainly in the epiligament. These groups showed no significant statistical difference (p = 0.194).

**Table 8:** Comparison of location of blood vessels among three groups with single injury based on duration of injury Group I: 2-5 months, group II: 6-8 months, and group III: 9-24 months. Freeman-Halton extension of the Fisher exact probability test was performed.

Location of blood vessels	Group I single injury (n = 6)	Group II single injury (n = 7)	Group III single injury (n = 6)	p-value
Epiligament	3	2	4	0.194
Midsubstance	3	1	1	
Both	0	4	1	

The predominant location of blood vessels between group I single and group I multiple injuries is given in Table 9. These two groups did not reveal any significant difference in the predominant location of blood vessels (p = 0.714).

**Table 9:** Comparison of location of blood vessels between group I single injury and group I multiple injuries Group I: 2-5 months. Freeman-Halton extension of the Fisher exact probability test was performed.

Location of blood vessels	Group I single injury (n = 6)	Group I multiple injuries (n = 4)	p-value
Epiligament	3	1	0.714
Midsubstance	3	2	
Both	0	1	

## Discussion

This study was conducted to ascertain the healing potential of the tibial remnant of ruptured ACL for its possible utility in the newly upcoming remnant-preserving ACL reconstruction surgeries. The results point toward and support the previous findings that ACL indeed has healing potential [7,15-17].

Blood vessel density

The median blood vessel density (blood vessels/sq. mm) in the tibial remnant of ruptured ACLs was found to be 5.50 (3.30, 10.23) per sq. mm in our study which appears to agree with other studies [15,16]. ACL was considered to be a relatively hypovascular structure [18]. The intact ACL studied by Murray et al. showed mean blood vessel densities of 1.5 per mm at the cut edge and 0.24 per mm farther from the cut edge [16]. The blood vessel density seen in ruptured ACL in the present study is higher than that of normal ACL reported by Murray et al. [16]. Similar observation was made in the previous studies [5,16,17]. Trocan et al. noticed mean blood vessel density to be 43 per 20x field in ruptured ACL and 15.2 per 20x field in intact ACL [5]. They observed angiogenesis in synovium in 83% of ruptured ACL and 20% of intact ACL. Angiogenesis inside ligament was seen in 35.18% of ruptured ACL and none of the intact ACL [5]. Murray et al. studied ruptured ACLs and characterized them into four phases. Inflammatory phase (<3 weeks) showed many inflammatory cells around the blood vessels [16]. Arterioles and venules were dilated. Capillaries were congested with thrombus in lumens. Epiligamentous regeneration (3-8 weeks) showed increased blood vessel density in epiligament, just below synovial layer. Proliferative phase (8-20 weeks) showed neovascularization in the collagen bundles of ligament with highest blood vessel density at 13/mm. Remodeling phase (52-104 weeks) showed reduction in blood vessel density at 2/mm [16]. Nayak et al. observed the mean blood vessel density to be reduced at 7-12 weeks, peaked (6.28 per sq. mm) at 13-20 weeks, later reduced (0.68 per sq. mm) at >50 weeks [15]. The comparison of the group-wise blood vessel densities of present study with previous studies is given below (Table 10). There was a decreasing trend of blood vessel density from first to second group similar to other studies [15,16]. However, the third group showed higher blood vessel density, unlike the previous studies.

**Table 10:** Comparison of group-wise blood vessel densities of present study with previous studies Nayak et al. [15], Murray et al[16]. IQR: interquartile range, SD: standard deviation.

Present study		Nayak et al.		Murray et al.	
Duration of injury	Blood vessel density (per sq. mm) (Median, IQR)	Duration of injury	Blood vessel density (per sq. mm) (Mean)	Duration of injury	Blood vessel density (per mm) (Mean ± SD)
2-5 months	5.43 (3.09, 11.77)	7-12 weeks	4.37	8-12 weeks	5.1 ± 3.1
		12-20 weeks	6.28	16-20 weeks	13.3 ± 4.9
6-8 months	4.80 (3.04, 9.34)	21-50 weeks	2.41	-	-
9-24 months	6.94 (3.84, 8.68)	> 50 weeks	0.68	52-104 weeks	2.1 ± 2.0

The comparison of blood vessel ranges suggests a trend of very high ranges (10.1-40 per sq. mm) of blood vessel density being more common in two to five months and six to eight months rather than at nine to 24 months, although it did not reach significance statistically. This finding however is similar to the previous studies indicating higher blood vessel density at first 20 weeks, reduced in 21-50 weeks, and drastically reduced blood vessel density at >50 weeks [15]. There was no significant difference between single and multiple injuries denoting no relation between blood vessel density and number of injuries. The densely cellular areas showed a higher number of blood vessels, confirming the conclusion of Sonnery-Cottet et al., that the blood vessel density was significantly higher in hypercellularity areas [19].

Luminal area

The luminal area studied showed the presence of capillaries alongside arterioles and venules. The coexistence of immature vessels lacking lumen and intermediary vessels with narrow lumen in the same section denotes angiogenesis as documented in earlier studies [5,7,15-17]. Angiogenesis has great importance in the healing process of ligaments [15]. Absence of angiogenesis is considered to be the chief factor for lack of healing in avascular tissues such as articular cartilages, specifically the inner zone [16]. The presence of angiogenesis, as seen in the present study, is crucial for newer treatment modalities to promote healing.

Location of blood vessels

Concerning the location of blood vessels in the ligament, Murray et al. have noticed the blood vessels in the epiligament during the epiligamentous phase of three to eight weeks [16]. After eight weeks, during the proliferative phase, the blood vessels started developing in the midsubstance. During the remodeling phase, between one and two years, the blood vessels in the midsubstance started diminishing [16]. We observed that 40% had predominant location in two to five months in epiligament; however, 50% had in midsubstance. As the duration progressed toward six to eight months, the blood vessels were equally present in epiligament and midsubstance in 50% and predominantly in epiligament in 37.5%. In nine to 24 months, the blood vessels were mainly in epiligament in 66.6% and diminished in midsubstance, as mentioned by a previous study [16]. However, less sample size was a limitation. This change of predominant location of blood vessels with duration of injury is critical to decide the timing for remnant-preserving ACL reconstruction surgeries.

Strengths of the study

ImageJ software was used for measuring the tissue section area to arrive at the blood vessel density and for measuring the luminal area of blood vessels. The entire slide was screened in the present study for the blood vessel density instead of counting only the vessel-rich areas of the slide as in previous study. Multiple fields were photographed for measuring luminal area of at least 10 vessels per slide, wherever feasible. Microphotographs were of high-quality images. The comparison of single and multiple injuries was not studied in the previous studies.

Limitations of the study

In the present study, the normal ACL could not be harvested for comparison from contralateral knee of the patient owing to ethical concerns, which stands as a limitation. Multiple injuries, being a very small cohort, has proved to be a challenge to find any significant difference from the single injury. Inflammatory (<3 weeks) and epiligamentous regeneration phases (3-8 weeks) could not be included due to the conventional surgical protocol of ACL reconstruction surgery which could influence the correlation of blood vessel density with duration of injury. Smaller sample size could have been a limitation to find significant difference among the groups based on duration of injury. Individual endothelial cells, as seen in immunohistochemistry, could not be detected by routine H&E staining.

## Conclusions

In summary, this study characterizes the histological features of ruptured ACL as blood vessel density, correlation with duration of injury, luminal area, and predominant location of blood vessels. The presence of immature and intermediary vessels denoted angiogenesis. More patients with earlier duration of injury showed a very high range (10.1-40 per sq. mm) of blood vessels compared to patients of later duration. Future studies correlating the functional outcome of patients with remnant preservation surgeries having higher blood vessel density would shed light onto the practical applications of these biological parameters.
